# Coincidental light chain induced proximal tubulopathy with lupus nephritis: a case report and review of the literature

**DOI:** 10.1186/s13256-021-02990-4

**Published:** 2021-08-04

**Authors:** Wael Mostafa Hamza, Ahmed Mohammed AlEssa

**Affiliations:** Department of Pathology and Laboratory Medicine, College of Medicine, King Fahd Hospital of the University, Imam Abdulrahman Bin Faisal University, Dammam, Saudi Arabia

**Keywords:** Proximal tubulopathy, Lupus nephritis, Plasma cell dyscrasia, Paraproteinemia, Free light chain

## Abstract

**Background:**

We report a case of light chain proximal tubulopathy associated with lupus nephritis in a patient known to have systemic lupus erythematosus. The kidney can be injured in several ways in any of these disorders. Light chain proximal tubulopathy is a rare form of renal tubular injury that may occur in and complicate plasma cell dyscrasia, characterized by cytoplasmic inclusions of the monoclonal light chain within proximal tubular cells. Lupus nephritis is a common form of renal injury as it occurs in about 25–50% of adult patients with systemic lupus erythematosus.

**Case presentation:**

We present a 57-year-old African patient known to have systemic lupus erythematosus and hypertension presented with a new complaint of microscopic hematuria. A renal biopsy was performed and revealed lupus nephritis class II concurrently associated with light chain induced proximal tubulopathy. A subsequent bone marrow biopsy was performed, which revealed multiple myeloma.

**Conclusions:**

We report a case of coincidental lupus nephritis and proximal tubulopathy featuring a combined constellation of rare histopathological features that might add to the relationship between systemic lupus and paraproteinemia.

## Introduction

Plasma cell dyscrasia (PCD) refers to a spectrum of several disorders characterized by monoclonal paraprotein production, with a wide variety of clinical presentations and outcomes [1]. The renal involvement in PCD is a common finding as it is present in up to half of myeloma patients at presentation [2]. Renal insufficiency is a defining event in patients with multiple myeloma. Myeloma cast nephropathy is the most common presentation or histologic finding and the second most common cause of death in these patients [1, 3]. Many studies have described renal lesions associated with PCD in detail [2, 4]. The renal involvement in multiple myeloma and related disorders is multifactorial, including indirectly through the effect of paraproteins over the glomeruli (light chain deposition disease), the proximal tubules (proximal tubulopathy), the distal tubules (myeloma cast nephropathy), and the renal vasculature (light chain amyloidosis), or rarely directly through the infiltration of the renal parenchyma by the neoplastic plasma cells. In patients with multiple myeloma, myeloma cast nephropathy most commonly represents lesion (approximately 30%) [3].

Light chain proximal tubulopathy (LCPT) is characterized by cytoplasmic monoclonal light chain inclusions within proximal tubular cells [5]. The structure of these inclusions had been categorized as crystalline and noncrystalline. However, the importance of this categorization and the treatment effect is unknown for these cases. The most important predictor of the disease progression is the early detection and the aggressiveness of treating the underlying etiology in some instances [5]. A review of the literature reveals 43 cases of light chain proximal tubulopathy with a presentation similar to our case. The kidney is commonly affected in a patient with systemic lupus erythematosus (SLE) as it occurs in approximately 25–50% of adult patients with systemic lupus [6] with different histopathological patterns and clinical manifestations. The involvement could be in the form of podocytopathy [7] or have one of the typical forms of lupus nephritis classes [8]. The coexistence of PCD or paraproteinemia in SLE patients has been described in the literature in very few cases with enigmatic pathogenesis and unsettled clinical features [9, 10]. On the other hand, patients with plasma cell dyscrasia might have autoimmune manifestations [11]. We report a case of coincidental lupus nephritis and LCPT. To the best of our knowledge, this is the first case to be reported.

## Case presentation

### Clinical data

A 57-year-old African patient was known to have SLE (diagnosed at the age of 27 years) and hypertension (diagnosed at the age of 52 years); both diseases were under control according to the patient’s provided history. The patient had been doing fine for the past 5 years and recently presented to the nephrology clinic at our hospital with new-onset microscopic hematuria. There is no known family history of SLE or other autoimmune diseases, or previous history of renal disease manifestations. Drug list at the time of presentation: tacrolimus 4 mg daily, prednisolone 15 mg daily, amlodipine 5 mg daily, valsartan 80 mg daily, nifedipine 20 mg daily, pantoprazole 40 mg daily, vitamin D3 1000 unit capsule daily, calcium carbonate 1500 mg daily, and Vitamin B complex one tablet daily. No definite data are available on the compliance of the patient with the reported medications as the patient was initially diagnosed for SLE and hypertension elsewhere. During the follow-up in our hospital (last 7 years), the patient was apparently compliant with regular filling of medications every 3 months. The laboratory findings and urine analysis at the time of presentation are listed in Tables 1 and 2, respectively.

**Table 1:** Laboratory findings at the time of presentation

Laboratory test	Result	Reference range
White blood cells	7.2 × 10^3^/μL	4.0–11.0 × 10^3^/μL
Red blood cells	3.8 × 10^6^/μL	4.2–5.5 × 10^6^/μL
Hemoglobin	9.4 g/dl	(12–16 g/dl)
Hematocrit	29.2%	(37–47%)
Mean corpuscular volume	76.9 fl	(80–94 fl)
Mean corpuscular hemoglobin	24.9 pg	(27–32 pg)
Mean corpuscular hemoglobin concentration	32.3 g/dL	(32–36 g/dl)
Serum creatinine level	1.23 mg/dl	(0.6–1.0 mg/dl)
Blood urea nitrogen	19 mg/dl	(7–18 mg/dl)
Urine protein	53 mg/dL (of tubular and glomerular origin)	(≤ 11.9 mg/dl)
24-hour urine protein	898 mg/24 hours	(50–80 mg/24 hours)
Sodium	136 mEq/L	(136–145 mEq/L)
Potassium	4.80 mEq/L	(3.5–5.1 mEq/L)
Chloride	103 mEq/L	(98–107 mEq/L)
Serum albumin	2.9 g/dl	(3.4–5.0 g/dl)
Total bilirubin	0.3 mg/dl	(0.2–1.2 mg/dl)
Direct bilirubin	< 0.05 mg/dL	(0.05–0.2 mg/dl)
Erythrocyte sedimentation rate	89 mm/hour	(0–20 mm/hour)
C3 complement	147 mg/dL	(90–180 mg/dl)
C4 complement	14 mg/dL	(10–40 mg/dl)
C-reactive protein	0.30 mg/dL	(0.05–0.3 mg/dl)

**Table 2:** Urine analysis result at time of presentation

Test	Result	Reference range
Color	Yellow	–
Clarity	Light turbid	–
Glucose	Negative	Negative
Bilirubin	Negative	Negative
Ketone	Negative	Negative
Specific gravity	1.019	(1.00–1.030 1.010 to ≥1.030)
Blood	Present (small amount)	Negative
pH	5	(5.5–6.0)
Protein	15 mg/dl	Negative
Urobilinogen	Negative	(0.2–1.0)
Nitrate	Negative	Negative
Leukocytes	Large	Negative
White blood cell	> 200	0–2/high-power field
Red blood cell	2–5	0–3 /high-power field
Cast	None seen	Negative
Epithelial cells	Rare	(None-Rare)
Necrotic epithelial cells	None seen	None
Yeast cells	None seen	None
Mucus threads	None seen	None to rare
*Trichomonas*	None seen	None
Bacteria	+1	None to rare
Spermatozoa	None seen	None
*Schistosoma*	None seen	None

A renal biopsy was performed.

### Histopathological examination

**Fig. 1:**
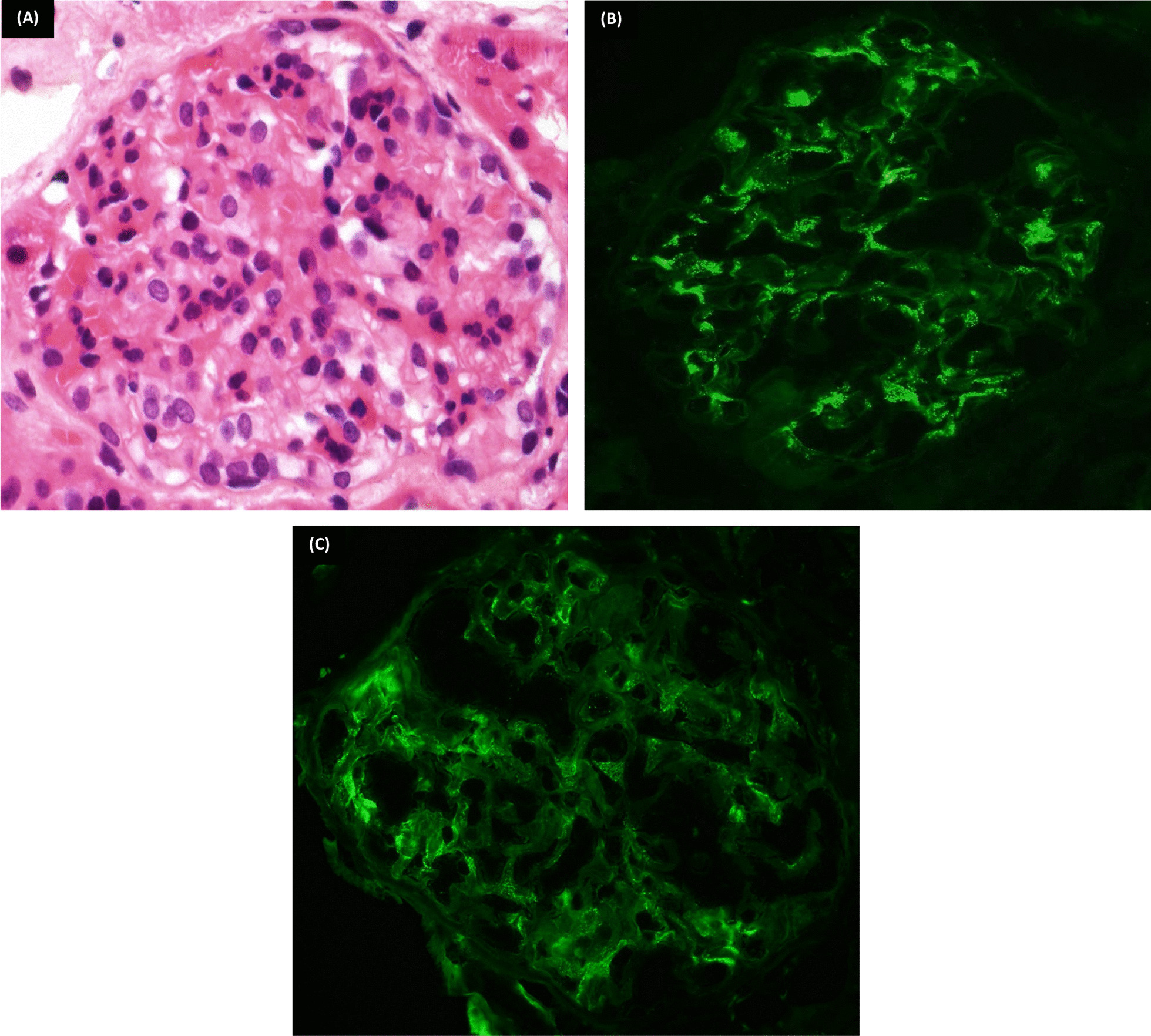
Glomerular findings (by LM and IF). **A** [Hematoxylin and eosin (H&E) ×400]: A glomerulus showing expanded mesangial matrix associated with mesangial hypercellularity. **B** [Immunofluorescence immunoglobulin G (IF-IgG) ×400]: Positive mesangial granular deposits; score 2. **C** (IF-IgA ×400): Positive mesangial granular deposits; score 1

**Fig. 2.**
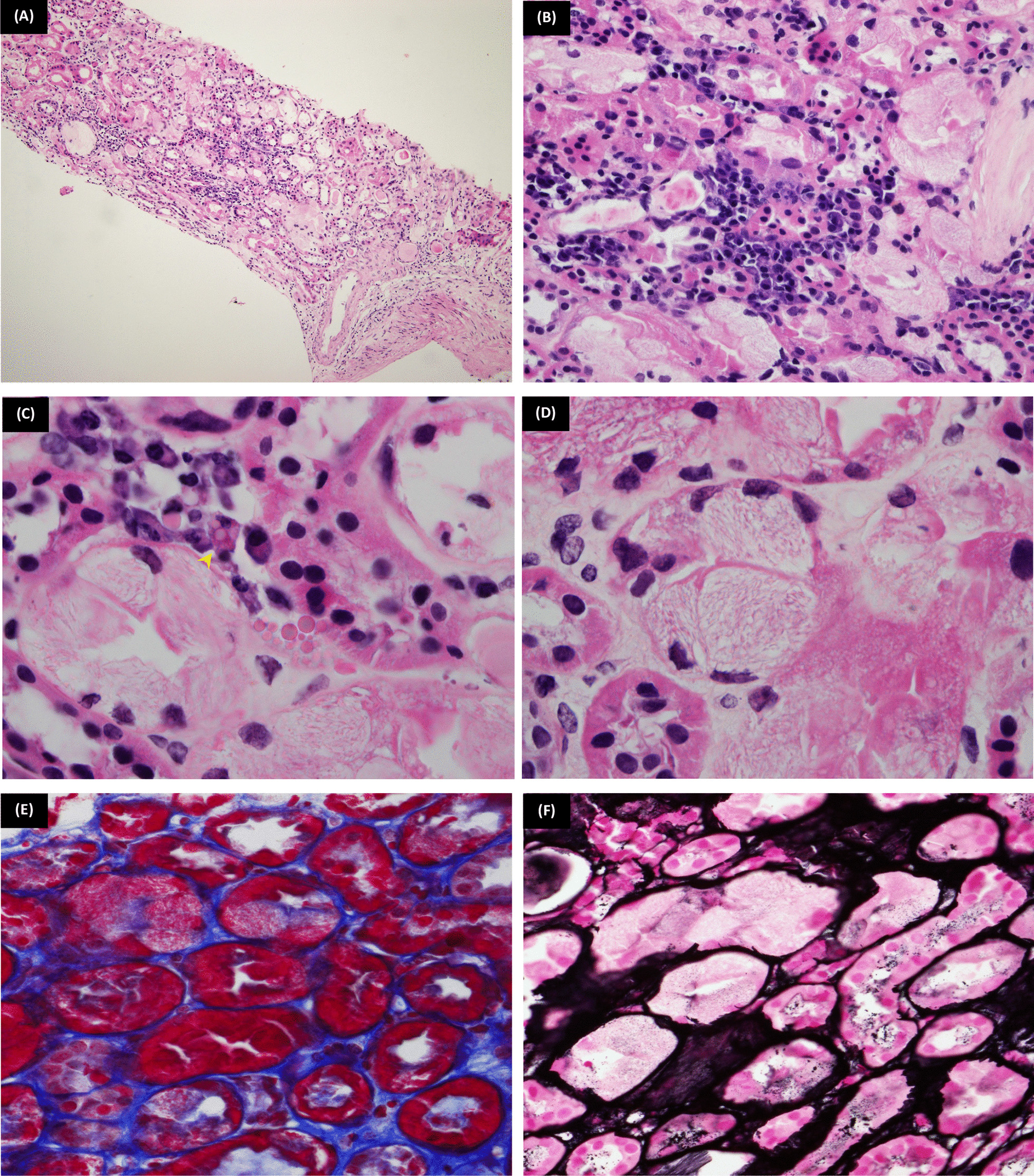
Proximal tubular lesions. **A** (H&E ×100): Renal cortical tissue showing mononuclear (plasmacytic infiltrate) and apparently tubular cytoplasmic inclusions. **B** (H&E ×200): The tubular epithelium showing textured intracytoplasmic inclusions. **C** (H&E ×1000): Monoclonal plasma cells; a Mott cell is seen “arrow head.” **D** (H&E ×1000): The proximal tubular epithelium showing textured fibrilloid inclusions of monoclonal light chains. **E** (Trichrome ×400), **F** [Jones' Methenamine Silver (JMS) ×400]: The tubular inclusions are fuchsinophilic and silver negative

**Fig. 3.**
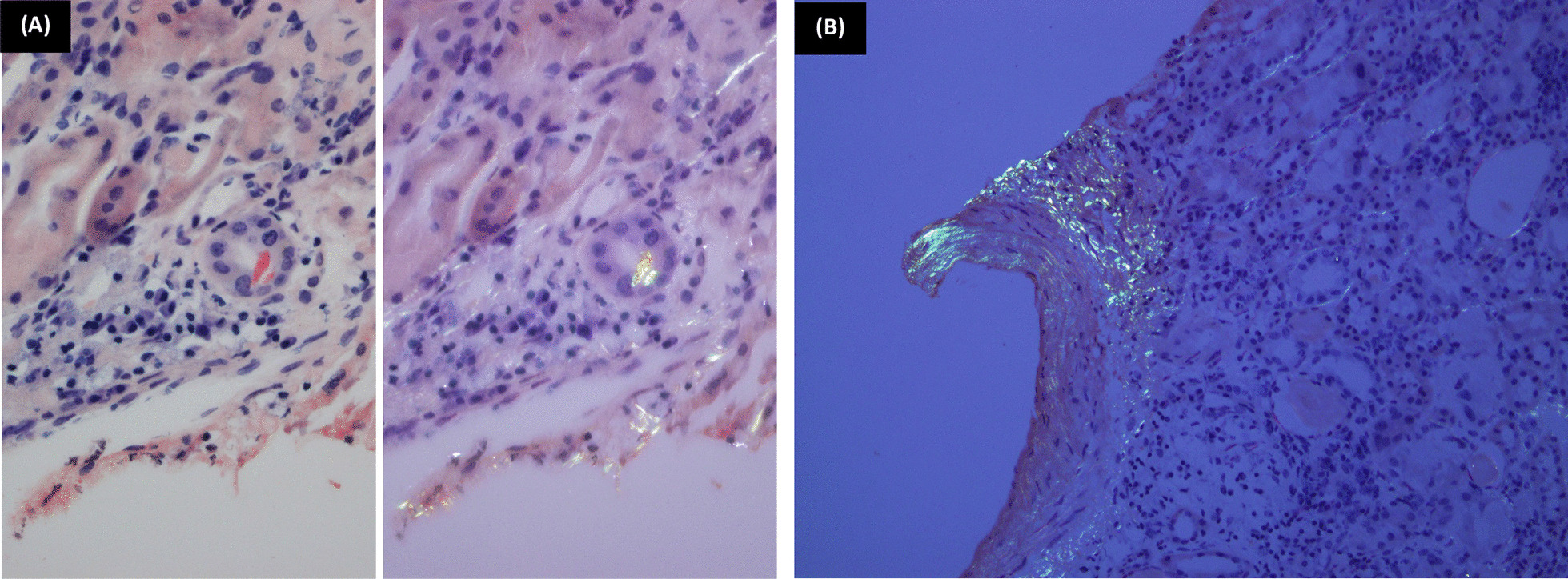
Amyloidal cast and deposits. **A** (Congo red ×200): Single orangeophilic cast seen giving apple-green birefringence under polarized light. **B** (Congo red ×200): An interlobular sized artery showing amyloidal deposits, as well as the adjacent interstitium

The light microscopic examination of the specimen revealed cores of renal corticomedullary tissue. Thirty-seven glomeruli were identified. The tufts show a mesangioproliferative pattern (Fig. 1A). The proximal tubular segments showed prominent cytoplasmic textured inclusions (Fig. 2); a single Congo red positive cast was detected (Fig. 3A). The interstitium shows patchy mononuclear predominantly plasmacytic infiltrate associated with edema and minimal loose fibrosis. Focal minimal interstitial deposit of amyloid material was detected. The arteries showed moderate-to-marked intimal fibroplasia and focal deposit of amyloid material (Fig. 3B). The arterioles showed focal mild hyalinosis.

Immunohistochemical staining by immunofluorescence (IF): two glomeruli presented in the IF submitted sample showed glomerular mesangial deposits for IgG (score 2+) and IgA (score 1+) (Fig. 1B, C). No immune deposits for IgM, C3, or C1q were revealed.

**Fig. 4.**
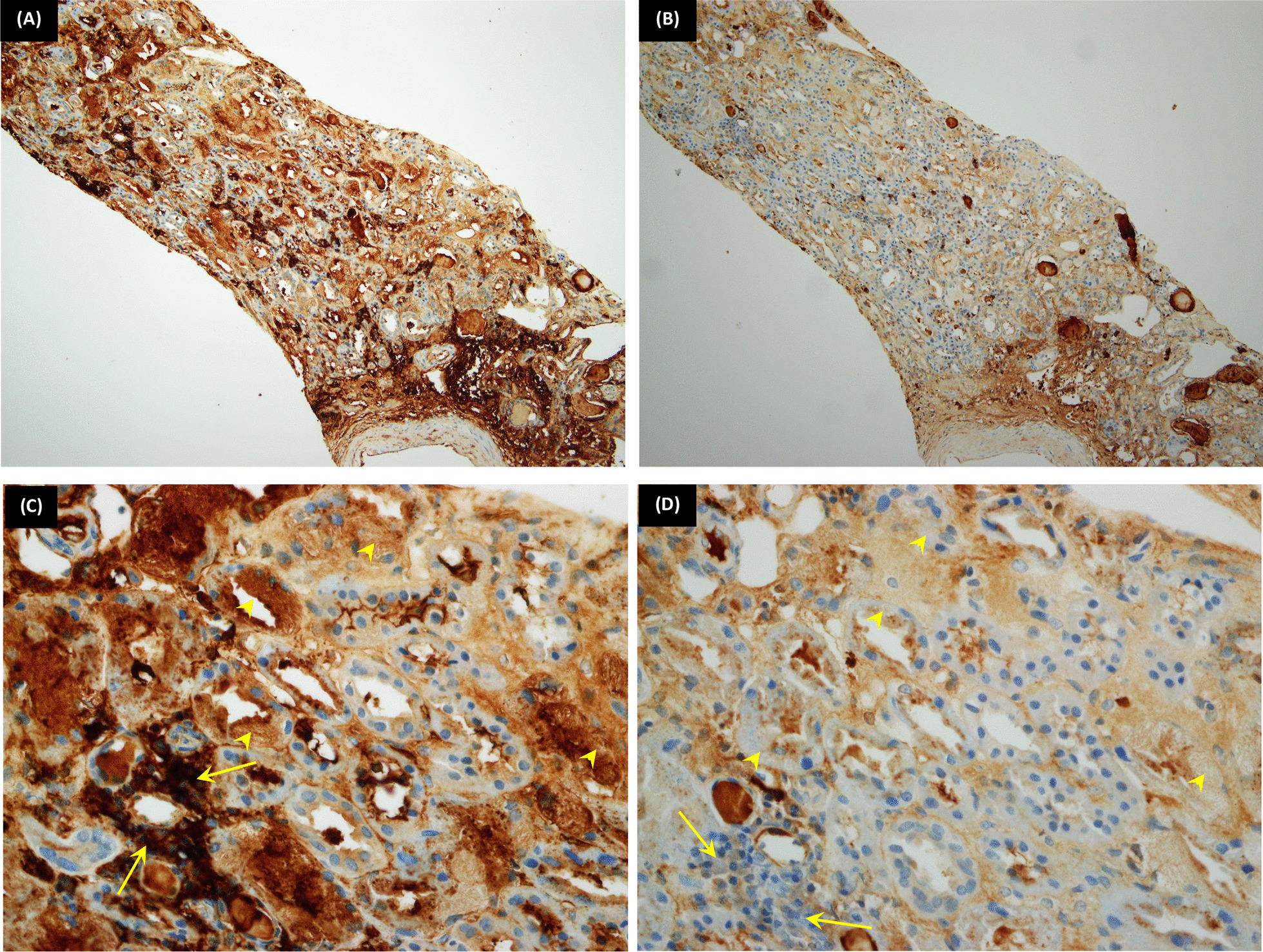
Immunoperoxidase stains (kappa and lambda); tubular findings. **A**, **B** (Immunoperoxidase technique-A: Kappa light chain, B: Lambda light chain ×100): The same core as in Fig. 2A; the plasmacytic infiltrate and inclusions showing positivity for kappa light chains rather than lambda. **C**, **D** (IP-C: Kappa light chain, D: Lambda light chain ×200): The arrowheads are pointing to intratubular cytoplasmic inclusions that are positive for Kappa (Fig. 2C) and negative for Lambda (Fig. 2D). The arrows are pointing to interstitial plasmacytic infiltrate that is also positive for Kappa (Fig. 2C) and negative for Lambda (Fig. 2D)

By immunoperoxidase technique (IP) (Fig. 4): the kappa light chain stain showed strong cytoplasmic positivity within proximal tubular segments and aggregates of plasma cells. The lambda light chain stain showed weak focal positivity.

Electron microscopy (EM) specimen was composed of fibrous tissue and skeletal muscle fibers.

A diagnosis of lupus nephritis class (IIa), associated with light chain induced proximal tubulopathy, minimal interstitial and vascular amyloidal deposits, and hypertensive renovascular changes was made.

### Further action

The clinician decided to perform serum protein electrophoresis. The results are presented in Table 3.

**Table 3:** Serum protein electrophoresis results

Total protein	8.8 g/dl	(6.4–8.2 g/dl)
Albumin	3.4 g/dl	(3.2–5.0 g/dl)
Alpha 1	0.2 g/dl	(0.1–0.4 g/dl)
Alpha 2	1.2 g/dl	(0.6–1.0 g/dl)
Beta	1.0 g/dl	(0.6–1.3 g/dl)
Gamma	3.1 g/dl	(0.7–1.5 g/dl)
Interpretation	Elevated gamma globulin with a spike in the gamma region (IgG 3.1 gm/dl) is compatible with monoclonal gammopathy suggests 24-hour urine for Bence Jones protein	

The 24-h Bence Jones protein analysis result was positive for Bence Jones protein, and the Bence Jones protein type was free kappa light chain. Bone marrow biopsy was subsequently performed and revealed hypercellular marrow showing aggregates of closely packed monoclonal plasma cells comprising more than 15% of the marrow cellularity.

The patient was advised to seek medical advice at another center for further radiological workup and treatment owing to unavailability of specified drugs at our hospital.

## Discussion

The renal pathology in PCD has several faces of involvement with different patterns and nephron topographic affection via free light chains. The tubular loop of the nephron is variably affected depending on whether the free light chains exert epithelial injury and where these light chains wield their effect (that is, proximal and distal tubulopathy). Interestingly, cases were reported involving both proximal and distal tubules [12, 13], and moreover, others recently described an associated glomerular tuft involvement [14, 15].

LCPT is relatively the latest tubular pattern described in this category, results from the accumulation of free light chains within the proximal tubular epithelia, and presents a diagnostic challenge not to be overlooked during daily practice. The accumulated inclusions are reported to be crystalline or noncrystalline and occasionally Congo red positive [16].

While the light microscopic pattern in our case was textured fibrilloid inclusions, in contrast to the variable light microscopic patterns that are described in the literature, including vacuolization with eosinophilic granules [14], small round amorphous intracytoplasmic bodies [16], osmotic diuresis like pattern [17], and marked vacuolization with the formation of apical blebs [18].

In this report, we used the immunoperoxidase technique on paraffin-embedded tissue to detect the light chains, with very satisfactory results. In most of the reported cases, the used technique is immunofluorescence, while immunoperoxidase [19, 20] and immunoelectron techniques [14, 17] are less frequently used for this purpose. Both kappa [14, 15, 17, 19] and lambda free light chains [12, 13, 16, 18] are described.

This case is unique in that there is infiltration of the kidney by monoclonal plasma cells, which is an exceedingly rare lesion to be detected in cases of multiple myeloma [21], whether associated with indirect nephropathic effect or not. Moreover, to the best of our knowledge, the detection of Mott cells described in this report is the first in the literature.

Another important feature, in this case, is the presence of a Congo red positive cast, a feature that could be rarely seen with cast nephropathy and not reported with LCPT, to our knowledge. Furthermore, we had vascular and focal interstitial amyloidal deposits in our biopsy that could be intuitively free light chain-induced or related to the chronicity of the autoimmune disease the patient had (a matter unsolved by immunostains owing to specimen limitation).

In our patient, plasma cell myeloma was confirmed by bone marrow biopsy results fulfilling the International Myeloma Working Group criteria (serum monoclonal protein ≥ 3 g/dL with positive urinary monoclonal (Bence Jones) protein/24 hours and clonal bone marrow plasma cells of more than 15%, in addition to presence of anemia with hemoglobin level 9.4 g/dL and renal disease manifestations) [1]. The presence of monoclonal marrow plasmacytosis in cases of LCPT is reported in several reports [12, 16, 19, 20], while others are negative for this feature [14, 15, 17]; a variable magnitude of plasma cell recruitment that reflects the diversity of the underlying plasma cell lesions.

SLE has been reported with various forms of plasma cell dyscrasia, including MGUS and amyloidosis. However, multiple myeloma is very rarely reported with SLE, that is, in just a couple of cases [9, 10].

Our case showed two biopsy-proven coincidental lesions: a glomerular lesion consistent with lupus nephritis class II and a tubular lesion of light chain induced tubulopathy that presented in an SLE-known patient without any previously known history of renal involvement. Both lesions are known to be mediated through abnormal activation of immune mechanisms, and although several hypotheses have been raised to explain the activation of monoclonal B cells in SLE patients, whether this coincidence has a causal relationship or just a fluke is still unresolved.

## Conclusion

We report a case of coincidental lupus nephritis and proximal tubulopathy featuring a combined constellation of rare histopathological features that might add to the relationship between systemic lupus and paraproteinemia.

## Data Availability

All the data utilized in the case report are available from the corresponding author.

## Abbreviations

SLE: Systemic lupus erythematosus
PCD: Plasma cell dyscrasia
LCPT: Light chain proximal tubulopathy
IF: Immunofluorescence
IgG: Immunoglobulin G
IgA: Immunoglobulin A
IgM: Immunoglobulin M
C3: Complement component 3
C1q: Complement component 1q
IP: Immunoperoxidase technique
EM: Electron microscope
MGUS: Monoclonal gammopathy of undetermined significance
